# Indiscriminate ssDNA cleavage activity of CRISPR-Cas12a induces no detectable off-target effects in mouse embryos

**DOI:** 10.1007/s13238-021-00824-z

**Published:** 2021-04-01

**Authors:** Yu Wei, Yingsi Zhou, Yajing Liu, Wenqin Ying, Ruiming Lv, Qimeng Zhao, Haibo Zhou, Erwei Zuo, Yidi Sun, Hui Yang, Changyang Zhou

**Affiliations:** 1 Institute of Neuroscience, State Key Laboratory of Neuroscience, Key Laboratory of Primate Neurobiology, Center for Excellence in Brain Science and Intelligence Technology, Chinese Academy of Sciences, Shanghai, 200031 China; 2 University of Chinese Academy of Sciences, Beijing, 100049 China; 3 Shanghai Center for Brain Science and Brain-Inspired Intelligence Technology, Shanghai, 201210 China; 4 Center for Animal Genomics, Agricultural Genome Institute at Shenzhen, Chinese Academy of Agricultural Sciences, Shenzhen, 518124 China

**Dear Editor,**

Newly discovered characteristics like “collateral effect” or trans-cleavage in CRISPR-Cas13 and CRISPR-Cas12 systems have enabled their usage in nucleic acid detection (Gootenberg et al. 2017, 2018; Chen et al. 2018). The collateral RNA cleavage of Cas13a has been reported to be harmful for cell development (Wang et al. 2019; Buchman et al. 2020). As a representative gene editor of CRISPR-Cas12 system, CRISPR-Cas12a (Cpf1) holds great potential for therapeutic applications in the future (Zetsche et al. 2015; Koo et al. 2018; Campa et al. 2019). However, when used for genome editing in mammalian cells, target-activated Cas12a has the risk to cleave transiently exposed ssDNA during replication, transcription and homology-directed repair processes (Chen et al. 2018) (Fig. 1A), raising the concern of its therapeutic applications. Therefore, the potential off-target effects caused by the indiscriminate ssDNA cleavage activity of Cas12a need to be carefully investigated.

**Figure 1:**
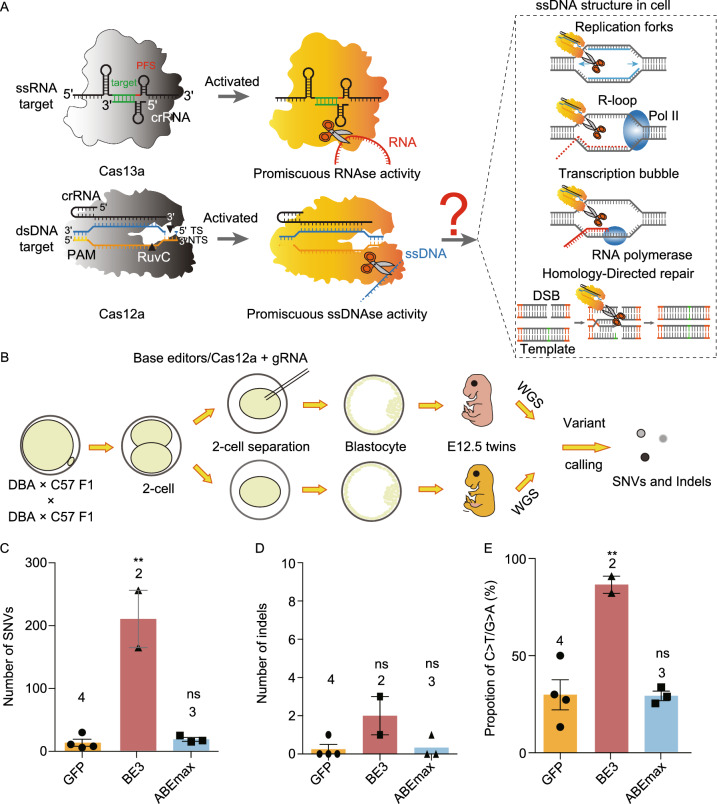
**GOAT detects off-target effects induced by BE3, ABEmax and indiscriminate ssDNA cleavage activity of CRISPR-Cas12a.** (A) “Collateral effect” or trans-cleavage in CRISPR-Cas13 and CRISPR-Cas12 systems. DNA replication, transcription, homology-directed repair and R loop structure would lead to the unwinding of double-stranded DNA (dsDNA) to ssDNA. Whether the indiscriminate ssDNA cleavage activity of Cas12a would induce genome-wide off-target effects in mammalian cells needs to be explored. (B) Experimental design of GOAT mediated genome-wide off-target detection. (C) Number of SNVs identified in GFP, BE3, and ABEmax injected groups. (D) Number of indels identified in GFP, BE3, and ABEmax injected groups by WGS. (E) The proportion of G·C to A·T mutations in GPF-, ABEmax-, and BE3-injected groups. Numbers above the columns represent the number of samples. *n* = 4 for GFP, *n* = 3 for BE3 and *n* = 3 for ABEmax groups. All values are presented as mean ± SEM. *P < 0.05, **P < 0.01, ***P < 0.001, ns, *P* ≥ 0.05, unpaired *t*-test

Recently, we developed a new approach called GOTI (Genome-wide Off-target analysis by Two-cell Injection) to detect off-target effects without the interference of single-nucleotide polymorphisms (SNPs) in individuals (Zuo et al. 2019). In this study, we designed an optimized method called genome-wide off-target analysis by twin blastomeres (GOAT) for off-target edits detection. Briefly, mouse embryos were separated into two embryos at two-cell stage, and then gene editing tools such as BE3, ABEmax and Cas12a were injected into one of the twin embryos (Figs. 1B and S1A). To increase the pregnancy efficiency, twin embryos were co-transferred with two ICR embryos to the pseudopregnant mouse. When the twin embryos developed to embryonic day 12.5 (E12.5), twin embryos and ICR embryos were distinguished by their eye colors and a SNP site on *Tyr* gene (Fig. S1B and S1C). The edited embryo was distinguished from the unedited twin embryo by high editing efficiency to induce indels and nucleotide substitutions on the target sites. Whole-genome sequencing (WGS) was performed on the genomic DNA of twin embryos, separately. Then single-nucleotide variants (SNVs) and indels were called in the injected sample, with its twin un-injected one as the reference (Figs. 1B and S1A). GOAT could distinguish the injected and un-injected embryos directly, while GOTI relies on massive FACS to separate edited cells from unedited cells. In addition, GOAT could also avoid the leak of two-cell injection, false-negative FACS sorting and inferior developmental competition ability of the injected blastomere.

To test the effectiveness of GOAT system, we included three groups in our study: GFP, BE3, and ABEmax groups (Fig. S1A). The developmental rate of twin embryos to blastocysts was more than 90%, and twin embryos developed to E12.5 was 23.0% ± 3.1% (*n* = 5; Table S1). WGS was conducted separately for the twin embryos at an average depth of 30 to confirm on-target editing efficiency and analyze the potential genome-wide off-target effects (Table S2). The activities of BE3 and ABEmax were confirmed by the high on-target efficiencies to introduce nucleotide substitutions (Figs. S1D, S2 and S3).

For the off-target effects, we found 14 SNVs and 0 indel per embryo on average in the GFP-injected group (Figs. 1C, 1D, S4 and Tables S3, S4, S5). For the BE3-injected embryos, we found 210 SNVs per embryo on average, 15 times more than those of the GFP group (*P* = 0.0025; Figs. 1C, S4 and Tables S3, S6). By contrast, indels showed no differences between BE3 and GFP groups (Fig. 1D). We observed that about 86% of SNVs were mutated from C to T, or G to A (Figs. 1E and S5), consistent with the results of GOTI method (Zuo et al. 2019). We also analyzed the off-target effects of ABEmax using GOAT. An average of 18 SNVs and 0 indel were detected in each embryo, similar to the number found in the GFP-injected group (*P* = 0.57; Figs. 1C, 1D, S4 and Tables S3, S4). Together, these results suggest that GOAT is a comparable approach to detect genome-wide off-target effects in mouse embryo comparing with GOTI.

We further used GOAT to analyze the genome-wide off-target effects of two commonly used Cas12a (LbCas12a and AsCas12a). Similarly, LbCas12a or AsCas12a mRNA and their crRNAs targeting *Dmd* or *Tp53* gene were injected into one of the twin embryos (Fig. 1B). The activities of LbCas12a and AsCas12a were confirmed by the high efficiency (100.0% ± 0.0% for LbCas12a-Dmd, 100.0% ± 0.0% for AsCas12a-Dmd; 83.3% ± 16.7% for LbCas12a-P53, 100.0% ± 0.0% for AsCas12a-P53; *n* = 3 twins for each group) to induce indels on *Dmd* gene. (Figs. 2A and S2). We found an average of 19 SNVs and 1 indel in LbCas12a group. Similarly, 18 SNVs and 1 indel were detected in AsCas12a group (Tables S3 and S4). Notably, the number of SNVs and indels of LbCas12a and AsCas12a groups were comparable to GFP ones and no significant difference was observed (Fig. 2B and 2C). For all the identified SNVs, the base substitution types showed no obvious bias (Fig. S5). Besides, the SNVs and indels observed in each embryo showed no overlap with those from other embryos (Fig. 2D). In contrast to the top predicted off-target sites, no similarity was observed between the adjacent sequences of detected off-target and the on-target sequences except for one off-target site (Figs. S3 and S6). Notably, this off-target sequence was previously reported as a crRNA-mediated off-target of *Tp53* (Kim et al. 2016)*,* demonstrating the sensitivity of GOAT method. We next analyzed the distribution of these SNVs in the genome context and found that they were randomly located in each chromosome, suggesting no preference for specific regions (Figs. 2E and S7). We further explored whether the SNVs and indels were enriched in transcription activated regions of the genome, where double-strand DNA (dsDNA) are frequently unwinded to ssDNA (Zhang et al. 2012), and found that no significant difference was observed between GFP and LbCas12a or AsCas12a groups (Fig. 2F and Table S7). These results indicated that the characteristics of mutations generated in LbCas12a and AsCas12a groups were consistent with those in GFP group. Since previous studies showed that Cas12a had the targeted-activated ssDNA cleavage activity *in vitro* (Chen et al. 2018; Li et al. 2018). We next applied the fluorophore quencher (FQ)-labeled reporter assays to investigate the correlation between the target DNA dosage and the percentage of cleaved ssDNA (Chen et al. 2018). Our results showed that when the target DNA was diluted to 10^−2^ nmol/L, the ratio of cleaved ssDNA was decreased to the control level (Fig. 2G). These experiments may explain that the low amount of targeted DNA in mammalian cells leads to a relatively small ratio of target-activated ssDNA cleavage activity of Cas12a, resulting in no detectable ssDNA cleavage induced off-target effects in mouse embryos.

**Figure 2:**
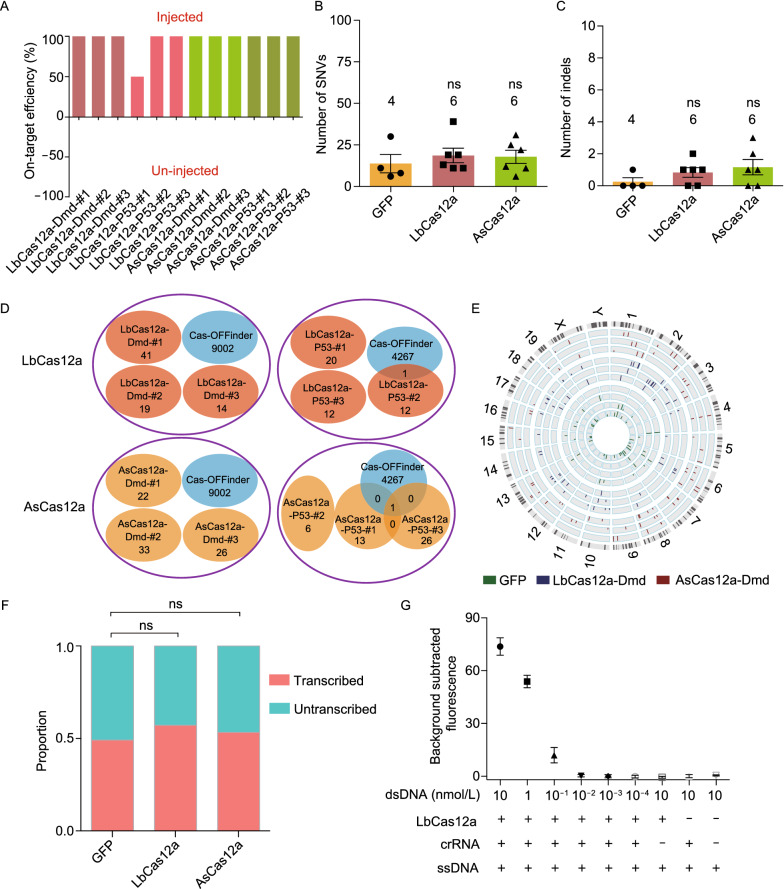
**On-target editing and off-target effects of LbCas12a and AsCas12a using GOAT.** (A) On-target editing efficiency identified by WGS for LbCas12a and AsCas12a groups. (B) Number of SNVs identified in LbCas12a and AsCas12a groups by WGS, where on-target editing was removed from the analysis. (C) Number of indels identified in LbCas12a and AsCas12a groups by WGS. (D) Overlap among SNVs and indels detected by GOAT with predicted off-targets by Cas-OFFinder. (E) Distribution of SNVs in the mouse genome in GFP, LbCas12a-treated and AsCas12a-treated samples. Embryos from inner circle to outer circle were GFP-#1, GFP-#2, GFP-#3, LbCas12a-Dmd-#1, LbCas12a-Dmd-#2, LbCas12a-Dmd-#3, AsCas12a-Dmd-#1, AsCas12a-Dmd-#2 and AsCas12a-Dmd-#3. (F) The distribution of off-target SNVs in the transcribed and un-transcribed regions. *P* values were calculated by Chi-square test on the number of SNVs in transcribed and un-transcribed regions of each group. (G) Correlation between the target DNA dosage and the presence of cleaved ssDNA. *n* = 4 twins for GFP, *n* = 6 twins for LbCas12a and *n* = 6 twins for AsCas12a groups; numbers above the columns represent the number of samples. All values are presented as mean ± SEM. *P < 0.05, **P < 0.01, ***P < 0.001, ns, *P* ≥ 0.05, unpaired *t*-test. Note that samples of the GFP group are also used in Fig. 1

In summary, we developed GOAT to detect genome-wide off-target effects of gene editing tools without the need of FACS. Compared with GOTI, GOAT was a simpler and lower cost method with comparable accuracy and sensitivity. Using GOAT analysis, we found that the trans ssDNA cleavage activity of Cas12a (LbCas12a and AsCas12a) was low in mouse embryos, suggesting that Cas12a is highly specific in mammalian cells. Our results further demonstrated that the target-activated, nonspecific ssDNA cleavage activity of Cas12a *in vitro* was induced by a large amount of targeted dsDNA. Recent studies reported that Cas13a-mediated targeting on massive copies of RNA generated substantial “collateral effect” in cultured cells and individual organisms (Wang et al. 2019; Buchman et al. 2020). By contrast, Cas12a only targeted a limited number of gene copies in mammalian cells, and thus would not cause broad ssDNA cleavage. Besides, protective DNA repairing mechanism can repair the limited number of ssDNA cleavage (Sancar et al. 2004). In addition, low-frequency trans cleavage off-target events and large scale deletions or insertions could be missed by our detection approach, resulting in the undetectable off-target effects in our study. Considering that LbCas12a and AsCas12a have comparable editing efficiencies, smaller size and lower mismatch tolerance comparing with spCas9, they hold great promise and competition for therapeutic application in the future (Kleinstiver et al. 2016).

## Electronic supplementary material

Below is the link to the electronic supplementary material.

(PDF 2442 kb)

(XLSX 11 kb)

(XLSX 11 kb)

(XLSX 30 kb)

(XLSX 34 kb)

(XLSX 24 kb)
