# Supplementary material for: Indiscriminate ssDNA cleavage activity of CRISPR-Cas12a induces no detectable off-target effects in mouse embryos
Source: Protein Cell. 2021 Apr 1;12(9):741–5. doi: 10.1007/s13238-021-00824-z (PMC8403096; doi:10.1007/s13238-021-00824-z)
Supplement: Supplementary file 1 — (PDF 2442 kb) [file 13238_2021_824_MOESM1_ESM.pdf]

Method

Mice

B6D2 F1 mice were used by crossing C57/BL6 female mice with DBA/2 male mice for superovulation and embryo collections. ICR female mice were used as pseudo-pregnant surrogate mother. The use and care of animals were carried out following the guideline of the Biomedical Research Ethics Committee of Shanghai Institutes for Biological Science, Chinese Academy of Sciences.

In vitro transcription (IVT) of gene editing tools

To generate IVT template of BE3, BE3 was amplified from plasmid with BE3-F and BE3-R primer pairs and T7 promoter at 5' end. After amplification, BE3 PCR products were gel-purified and used as IVT template. BE3 mRNA were in vitro transcribed using mMACHINE T7 ULTRA kit (Life Technologies) following the manufacture's instruction. BE4max mRNA, ABEmax mRNA, LbCas12a mRNA, AsCas12a mRNA and GFP mRNA were generated in the same way. Guide RNA used in the study were in vitro transcribed from PCR-generated template using MEGA shortscript T7 kit (Life Technologies). After in vitro transcription, both mRNA and sgRNA were purified with MEGA clear kit (Life Technologies) and eluted in RNase-free water. Guide RNA sequences and primers used in the study for generating IVT templates were listed as follows.

Guide RNA sequence

Guide RNA name	Sequence (5'-3')
BE3-*Tyr*-A	GGGTTTCTGCCTTGGCACAG
ABEmax-*Tyr*-B	ACCTCAGTTCCCCTTCAAAG
LbCas12a-Dmd	GTACCAGACCTCTTGAGAGA
AsCas12a-Dmd	GTACCAGACCTCTTGAGAGA
LbCas12a-P53	GCCACAGCGTGGTGGTACCT
AsCas12a-P53	GCCACAGCGTGGTGGTACCT

Primers for mRNA IVT

Name	Sequence(5'-3')
BE3 IVT F	TAATACGACTCACTATAGGG
BE3 IVT R	GTAATTGATTACTATTAATAACTAGCGGCCG
ABEmax IVT F	TAATACGACTCACTATAGGG
ABEmax IVT R	GTAATTGATTACTATTAATAACTAGCGGCCG
LbCas12a IVT F	TAATACGACTCACTATAGGGAGACCCAA
LbCas12a IVT R	CTTTCCGCCTCAGAAGCCATA
AsCas12a IVT F	TAATACGACTCACTATAGGGAGACCCAA
AsCas12a IVT R	CTTTCCGCCTCAGAAGCCATA
GFP IVT F	TAATACGACTCACTATAGGGCCACCATGGTGAGCAAGGG
GFP IVT R	TTACTTGTACAGCTCGTCCA

Primers for crRNA IVT

Name	Sequence(5'-3')
Tyr-A-F	TAATACGACTCACTATAGGG GGGTTTCTGCCTTGGCACAG GTTTTAGAGCTAGAAATAG
Tyr-A-R	GCACCGACTCGGTGCCACT
Tyr-B-F	TAATACGACTCACTATAGGGACCTCAGTTCCCCTTCAAAG GTTTTAGAGCTAGAAATAG
Tyr-B-R	GCACCGACTCGGTGCCACT
LbCas12a-Dmd-F	GAAATTAATACGACTCACTATAGGGAATTTCTACTAAGTGT AGATGTACCAGACCTCTTGAGAGAAATTTCTACTAAGTGTA GATGTACCAGACCTCTTGAGAGA
LbCas12a-Dmd-R	TCTCTCAAGAGGTCTGGTACATCTACACTTAGTAGAAATTTC TCTCAAGAGGTCTGGTACATCTACACTTAGTAGAAATTCCCT ATAGTGAGTCGTATTAATTTC
AsCas12a-Dmd-F	GAAATTAATACGACTCACTATAGGGTAATTTCTACTCTTGTA GATGTACCAGACCTCTTGAGAGATAATTTCTACTCTTGTAGA TGTACCAGACCTCTTGAGAGA
AsCas12a-Dmd-R	TCTCTCAAGAGGTCTGGTACTCTACAAGAGTAGAAATTATC TCTCAAGAGGTCTGGTACATCTACAAGAGTAGAAATTACCC TATAGTGAGTCGTATTAATTTC
LbCas12a-P53-F	GAAATTAATACGACTCACTATAGGGAATTTCTACTAAGTGT AGATGCCACAGCGTGGTGGTACCTAATTTCTACTAAGTGTA GATGCCACAGCGTGGTGGTACCT
LbCas12a-P53-R	AGGTACCACCACGCTGTGGCATCTACACTTAGTAGAAATTA GGTACCACCACGCTGTGGCATCTACACTTAGTAGAAATTCC CTATAGTGAGTCGTATTAATTTC
AsCas12a-P53-F	GAAATTAATACGACTCACTATAGGGTAATTTCTACTCTTGTA GATGCCACAGCGTGGTGGTACCTTAATTTCTACTCTTGTAGA TGCCACAGCGTGGTGGTACCT
AsCas12a-P53-R	AGGTACCACCACGCTGTGGCATCTACAAGAGTAGAAATTAA GGTACCACCACGCTGTGGCATCTACAAGAGTAGAAATTACC CTATAGTGAGTCGTATTAATTTC

Embryo collection, splitting and transplantation

Eight-week-old B6D2 F1 (C57BL/6 crossing with DBA/2 mice) female mice were superovulated by intraperitoneal injection of 0.75 IU PMSG on the first day and 0.75 IU hCG forty-eight hours after PMSG injection. Immediately after hCG administration, superovulated female mice were mated with 8-15 weeks old B6D2 F1 male mice. Zyogtes were collected from the mated mice oviducts 20 hours post hCG injection. After zygotes reached late 2-cell stage (46-49 hours post hCG injection),

twin blastomeres in each embryo were split into two individual blastomeres in a droplet of M2 (Millipore) medium containing 5 μg/ml cytochalasin B (CB). Briefly, zona pellicuda of the 2-cell stage embryo was partially ablated by lazer to remove one of the blastomere out of the 2-cell stage embryo while leaving another one in the same zona pellicuda with 45 μm diameter-wide needle. Removed blastomere was then transferred to vacant recipient zona pellicuda. In this way, two artificial twin embryos were generated for separate injection of mRNA and sgRNA afterwards. For injection, BE3 mRNA (50ng/μl), BE4max mRNA(50ng/μl), ABEmax mRNA(50ng/μl), LbCas12a mRNA (50ng/μl) or AsCas12a mRNA (50ng/μl) and sgRNA (50ng/μl) were co-injected into one of the artificial twin embryos by FemtoJet microinjector (Eppendorf) with constant flow settings. All the embryos were cultured in KSOM+AA (Millipore) at 37℃ and 5% CO2 until blastocyst stage for transplantation. The twin blastocysts were inspected for high quality before being picked for transferring to the oviducts of 0.5dpc pseudopregnant surrogate mice. In addition to the twin embryos, two E1.5 ICR embryos were co-transferred to improve pregnant efficiency.

Twin embryos collection and genotyping

Cesarean surgery was performed for surrogate mice at pregnancy day 12.5. After surgery, embryos were dissected out of yolk sac with tweezers removing extra-embryonic tissues and washed with PBS for 5-10 times in the dish. Each E12.5 embryo was divided into 2 parts, one part was used for Sanger sequencing to evaluate on-target editing efficiency and the other part was used for whole genome sequencing. Embryos were lysed by lysis buffer with proteinase K at 37℃ for 1h, and then incubated at 95℃ for 30min to inactivate the proteinase K. Genotyping PCR was performed for 35cycles at 95℃ for 30s, 60℃ for 30s and 72℃ for 1min, respectively. PCR products were analyzed by Sanger sequencing to differentiate the edited from unedited twin embryos. Genotyping primers were listed in the following table.

Primers used for genotyping

	Sequence(5’-3’)
Tyr-F	GATCTCTGATGGCCATTTTCCTC
Tyr-R	TTTTCTGCCCTGAGATATTATCAG
Dmd-F	TGTAGTTCACCCACAAGTATGTTGCT
Dmd-R	CATCTAGCTGGGCTTTACTTCAAAACA
Tp53-OF	TCACAGCACATGACGGAGGT
Tp53-OR	GCCCCTTCTCCCAGAGACTG
Tp53-IF	CGGAGGTCGTGAGACGCTGC
Tp53-IR	CTGCTGTTAAAGTAGACCCTGGG

Fluorophore quencher (FQ)-labeled reporter assays

30 nM LbCas12a (NEB, M0653S) was pre-assembled with 50 nM of *Dmd*-targeting crRNA in 37 ℃ for 10 min. Different concentration of dsDNA were added to the pre-assembled Cas12a and crRNA to incubate for 60 min in a 20 μL reaction system.

Then, 50nM FQ-Probe (TTATTATT flanked by 5' 6-FAM and 3' BHQ-1, Genscript) were added to the reaction system to incubate in a fluorescence plate reader (Flexstation 3) at 37 ℃ for 60 min. Fluorescence measurements were taken every 60 seconds (λex: 492 nm; λem: 518 nm).

Whole genome sequencing (WGS)

Twin embryos were collected from uterus of sacrificed surrogate female male for genomic DNA extraction. Genomic DNA was extracted using DNeasy blood and tissue kit (catalog number 69504, Qiagen) and twin embryo samples were sequenced at an average depth of 30x using 150bp paired-end Illumina X-Ten platform. Fastp (V0.20.0) were used to filter the low-quality reads with parameters '-q 20 -u 40 -M 0 -n 5 -1 80 -w 32'. Bwa-mem (0.7.16a) was used to align the clean reads to mm10 reference genome. (Li 2013) Samtools (1.6) was used to sort the mapped BAM files and GATK (4.0.12.0) was used to mark the duplicated reads. (Li et al. 2009; McKenna et al. 2010) Four major algorithms Strelka (2.9.x), Lofreq (v2.13), Mutect2 (v4.0.12.0) and Scalpel (v0.5.4) (Saunders et al. 2012; Wilm et al. 2012; Cibulskis et al. 2013; Fang et al. 2016) were used to identify the SNVs and indels. To reduce the computational burden, an optimized pipeline was used to identify the variants. Firstly, Strelka was used to indentify the genome-wide SNVs and Indels. The regions 200bp upstream and downstream of the variants identified by Strelka were treated as candidate regions. Secondly, Lofreq and Mutect2 were used to calculate SNVs of the candidate regions identified by Strelka, respectively. Scalpel and Mutect2 were used to identify the indels of the candidate regions, respectively. We also applied the CasOFFinder for the identification of potential sgRNA-dependent off-target SNVs and indels. The adjacent 400bp region of these variants were also used as candidate regions for indel detection by Scalpel. Only variants identified by all the three algorithms and with more than 10% allele frequencies were used for the following analysis. To strictly control the quality of the variants, we removed variants overlapped with UCSC repeat regions or reported in dbSNP (v140) database. Bedtools (v2.29.2) were used to perform the overlapping of variants (Quinlan and Hall 2010). Bam-readcount (V0.8.0) were used to calculate base frequency of the SNVs.

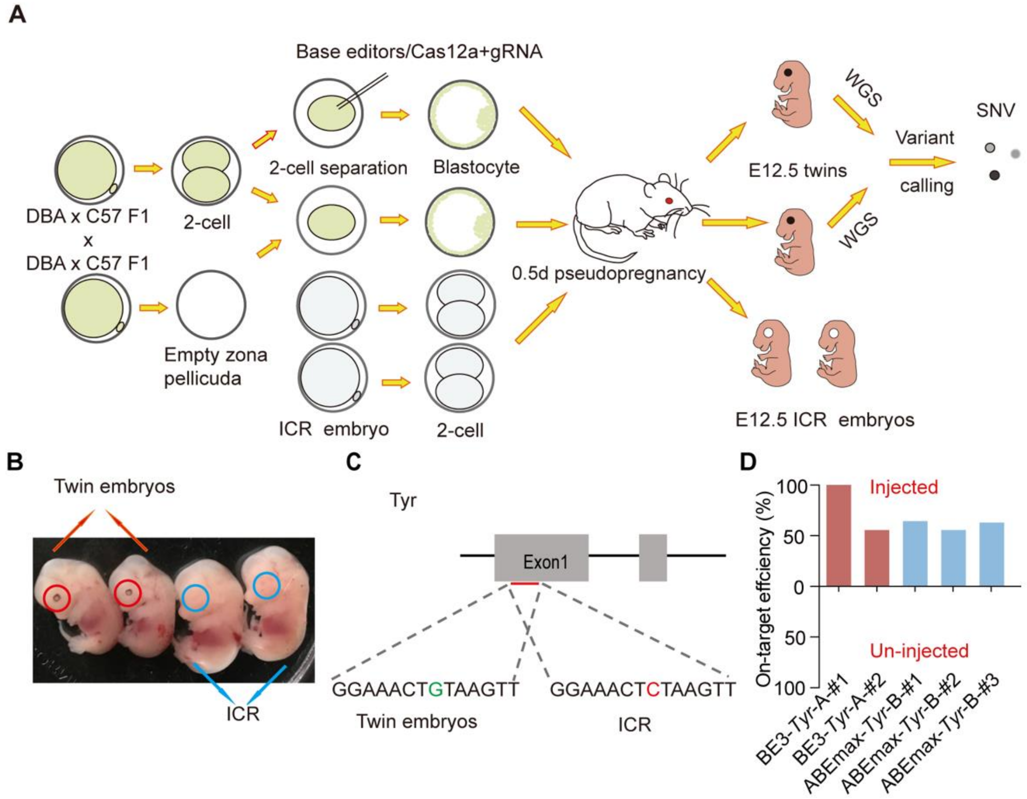

Figure S1. Overview of GOAT method and genome-wide off-target detection. (A) Scheme of experimental design. (B) A representative image showed E12.5 B6D2F1*B6D2F1 twin embryos (Left two embryos) with black eyes were distinguished from the co-transferred ICR embryos (right two embryos) with transparent eyes. (C) A SNP in *Tyr* gene was used to distinguish the twin embryos from the co-transferred ICR embryos. (D) On-target efficiency of BE3 and ABEmax identified by WGS. *Tyr*-A and *Tyr*-B represent different *Tyr* targeted sgRNAs.

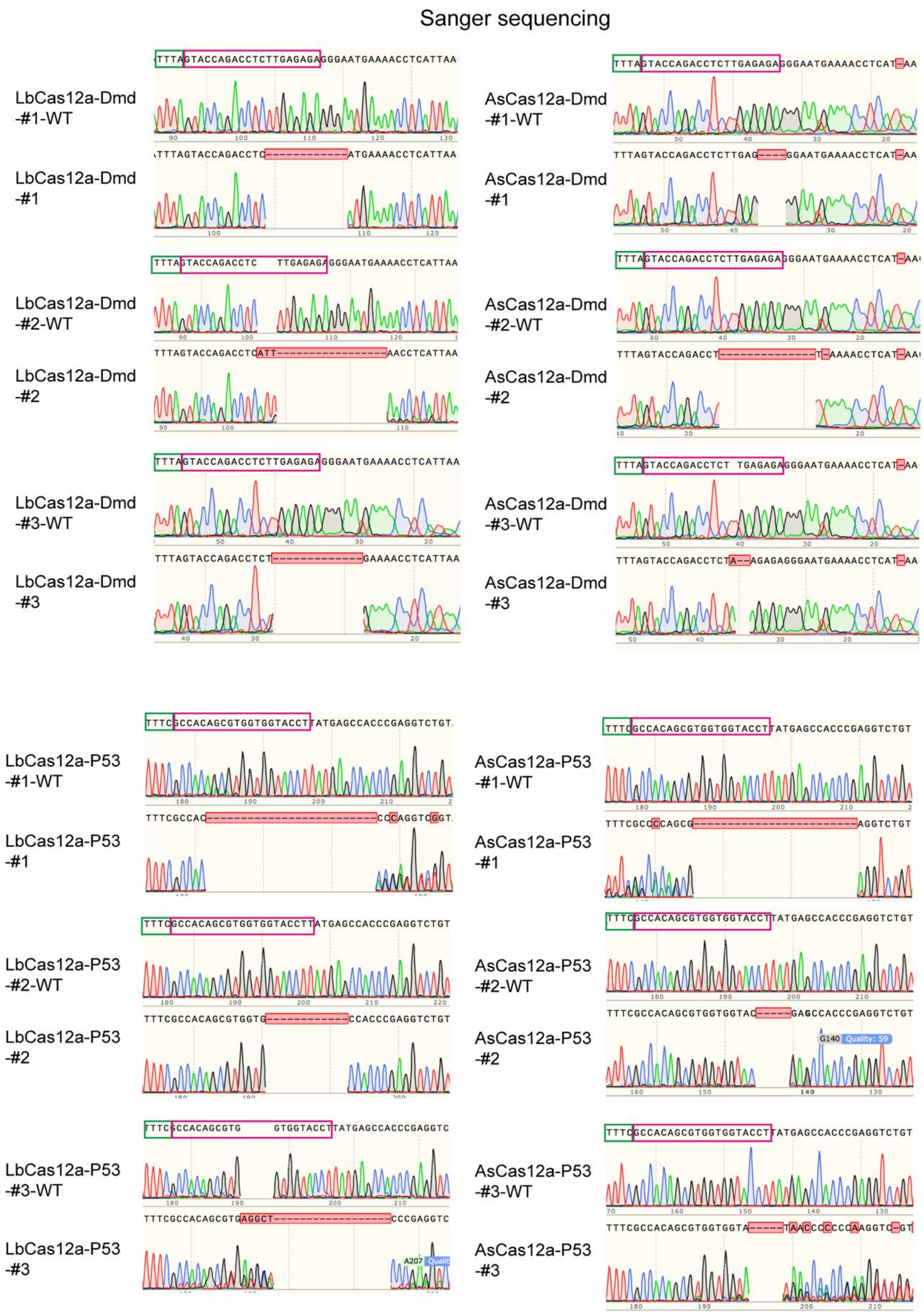

Figure S2. On-target efficiency from Sanger sequencing in injected and un-injected twin embryos. Sanger sequencing of Cas12a-injected and un-injected E12.5 twin embryos. Targeting sequences were marked with red rectangles and PAM sequences were marked with green rectangles.

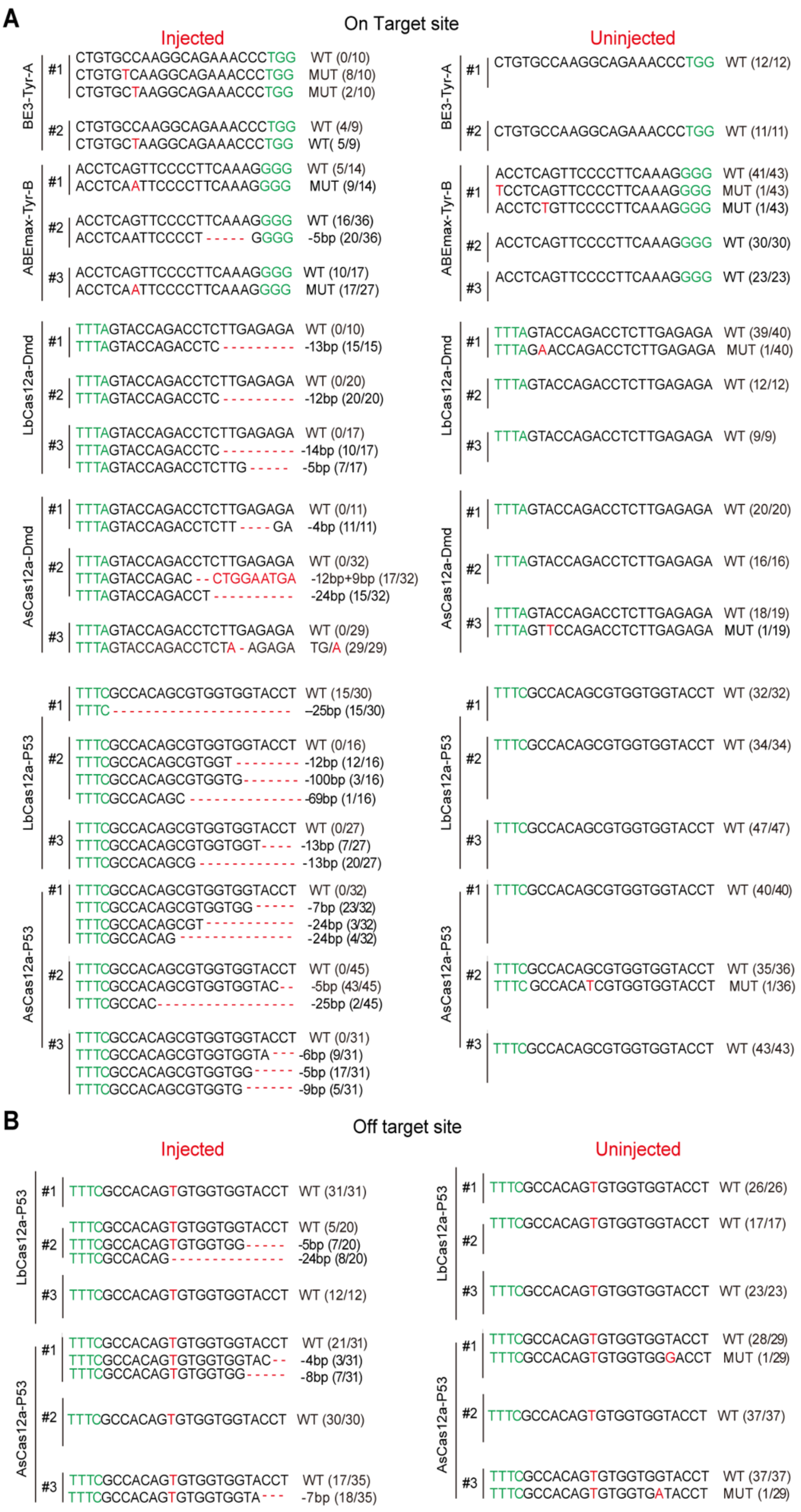

Figure S3. On-target and the predicated off-target sequences from WGS in injected and un-injected twin embryos. (A) On-target efficiency from WGS in injected and un-injected twin embryos. (B) The editing efficiency at the predicted TP53-mediated off-target site from WGS in injected and un-injected twin embryos.

The rare untargeted mutations in un-injected group were likely caused by sequence errors.

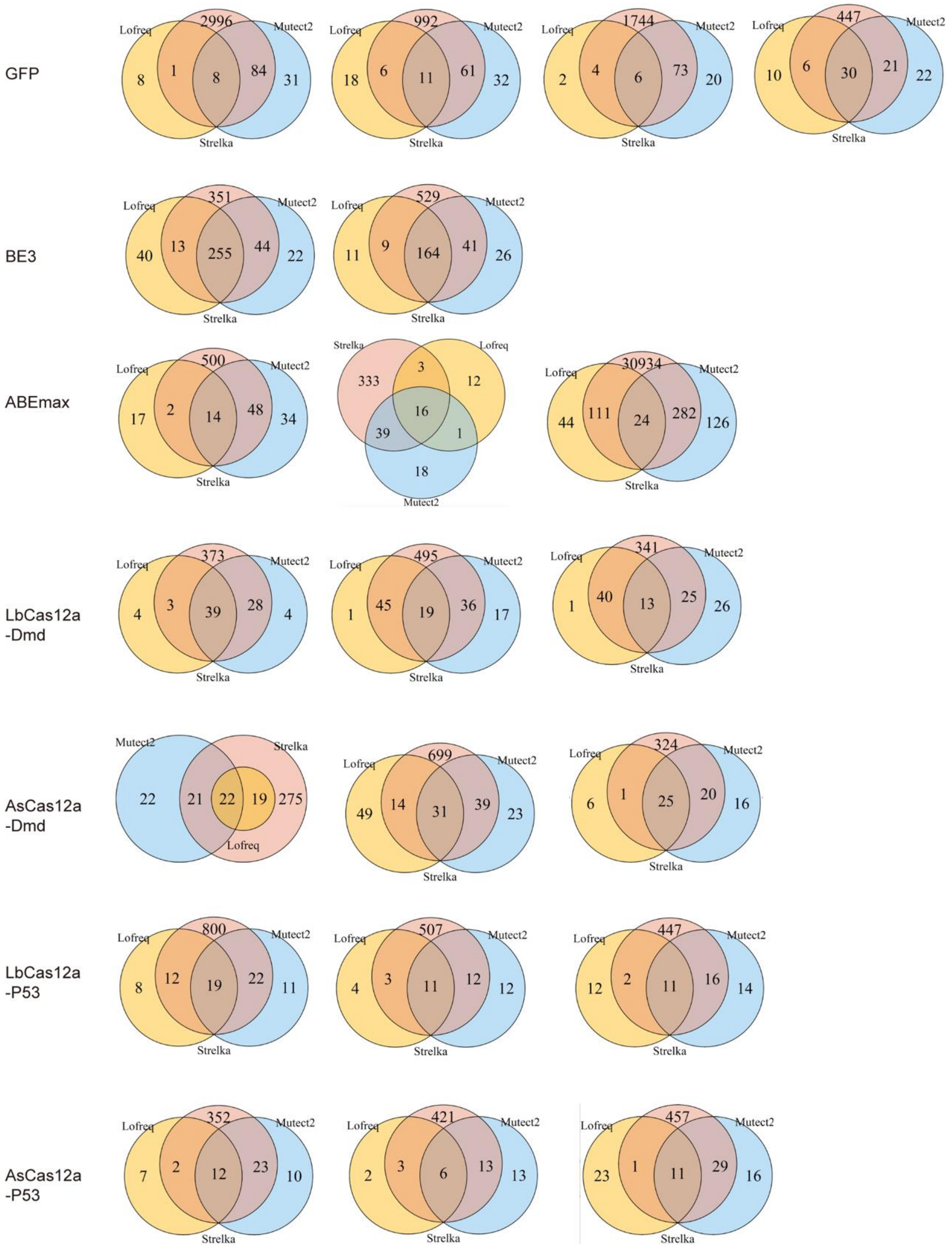

Figure S4. Venn diagrams of SNVs in each embryo analyzed by Lofred, Mutect2 and Strelka. SNVs were called by Lofred, Mutect2 and Strelka, separately. Common SNVs called by all the three algorithms were defined as true SNVs. The overlapping SNVs with allele frequencies less than 10% were removed from the following analysis.

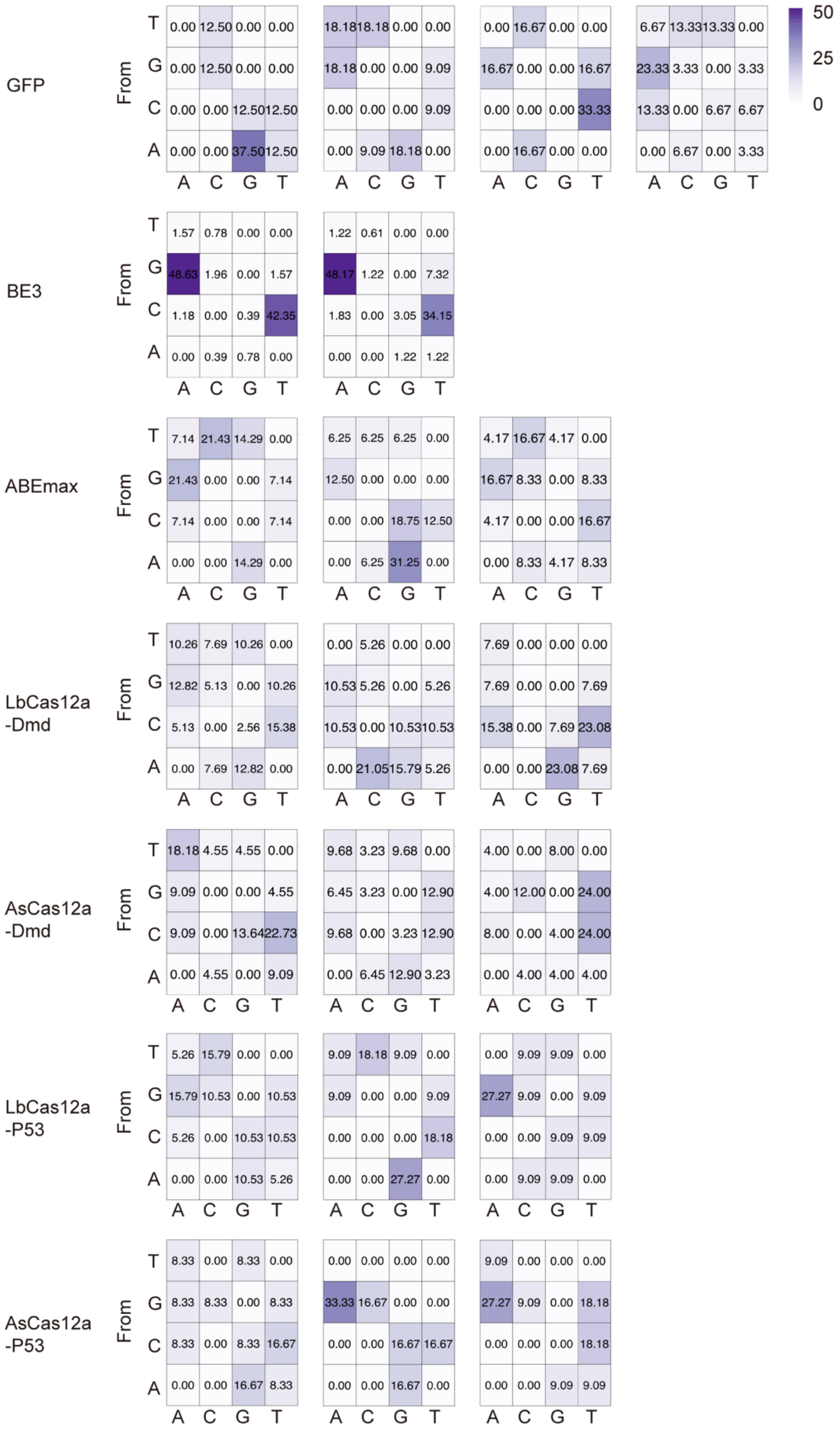

Figure S5. The distribution of base substitution types in each embryo. The number represents the percentage of a certain type of base conversion, and higher proportions are indicated by deeper colors.

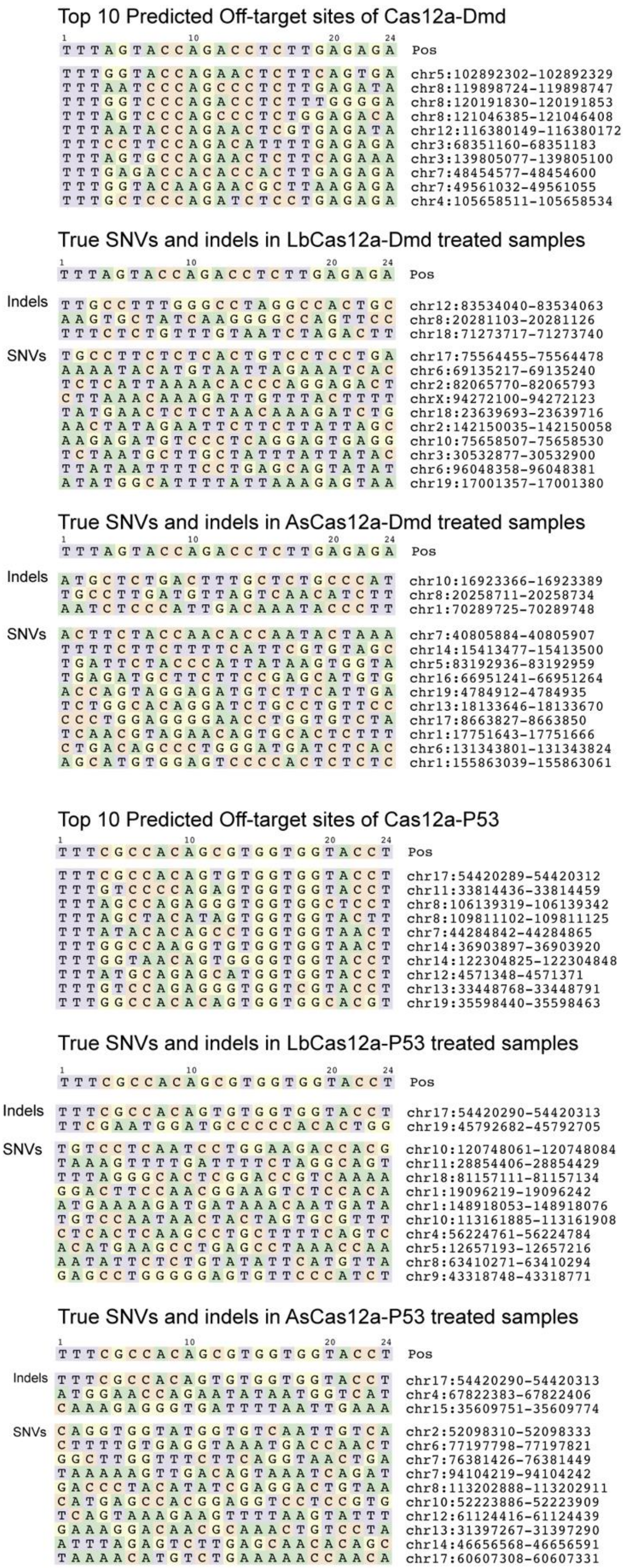

Figure S6. The sequence similarity between predicted off-target sites or identified SNVs/indels and on-target site. The top 10 predicted off-target sites were shown in the top panel, and adjacent sequences of the top 10 SNVs or indels in LbCas12a and AsCas12a groups were shown in the lower panels.

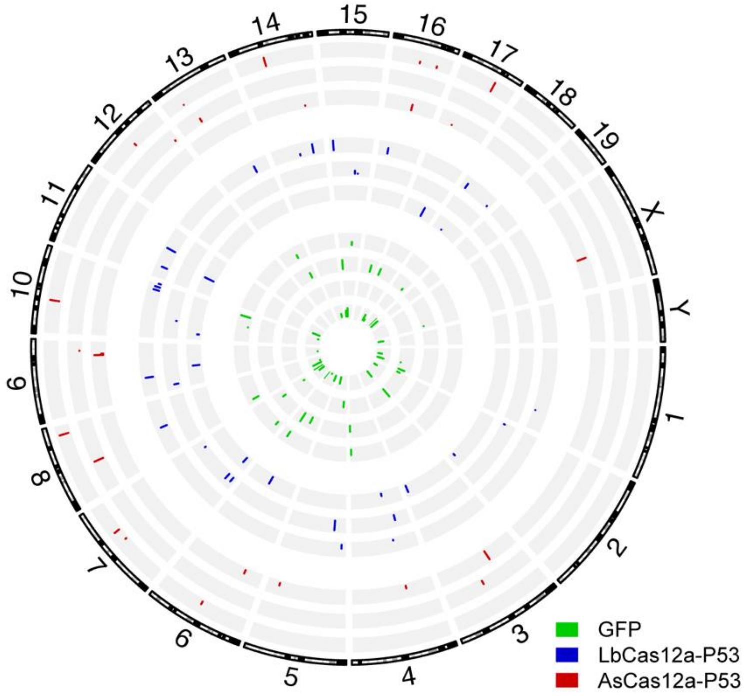

Figure S7. Distribution of SNVs in the mouse genome in GFP, LbCas12a-treated and AsCas12a-treated samples. Embryos from inner circle to outer circle were GFP-#1, GFP-#2, GFP-#3, LbCas12a-P53-#1, LbCas12a-P53-#2, LbCas12a-P53-#3, AsCas12a-P53-#1, AsCas12a-P53-#2 and AsCas12a-P53-#3. Note that samples of the GFP group are also used in Fig. 1.

Table S1. Development efficiency of the twin embryos in different injection groups.

Group	No. of embryos manipulated	No. of blastocysts (%)	No. of pairs of twin embryos transferred*	No. of pairs of E12.5 twin embryos obtained (%)
GFP	113*2	208/216 (96.3)	26	6/26 (23.1)
BE3	135*2	246/270 (91.1)	18	4/18 (22.2)
ABEmax	106*2	197/212 (92.9)	24	3/24 (12.5)
LbCas12a-Dmd	130*2	243/260 (93.5)	28	9/28 (32.1)
AsCas12a-Dmd	104*2	196/208 (94.2)	20	5/20 (25)
LbCas12a-P53	96*2	180/192 (93.4)	27	6/27 (22.2)
AsCas12a-P53	103*2	191/206 (92.7)	27	4/27 (14.8)

Each 2-cell stage embryo was separated into two blastomeres to get twin blastomeres.
*When the twin embryos developed to blastocyst stage, the well developing twin blastocysts were transferred to pseudopregnant mother.

Table S3. Summary of SNVs and indels identified from WGS in each embryo.

	GFP				BE3		ABEmax			LbCas12a-Dmd			AsCas12a-Dmd		
	1#	2#	3#	4#	1#	2#	1#	2#	3#	1#	2#	3#	1#	2#	3#
WGS SNVs	8	11	6	30	255	164	14	16	24	39	19	13	22	31	25
WGS indels	0	1	0	0	3	1	0	1	0	2	0	1	0	2	1

LbCas12a-P53			AsCas12a-P53		
1#	2#	3#	1#	2#	3#
19	11	11	12	6	11
0	1	1	1	0	3

REFERENCES

Cibulskis, Kristian, Michael S. Lawrence, Scott L. Carter, Andrey Sivachenko, David Jaffe, Carrie Sougnez, Stacey Gabriel, Matthew Meyerson, Eric S. Lander, and Gad Getz. 2013. 'Sensitive detection of somatic point mutations in impure and heterogeneous cancer samples', *Nature Biotechnology*, 31: 213-19.

Fang, Han, Ewa A. Bergmann, Kanika Arora, Vladimir Vacic, Michael C. Zody, Ivan Iossifov, Jason A. O'Rawe, Yiyang Wu, Laura T. Jimenez Barron, Julie Rosenbaum, Michael Ronemus, Yoon-ha Lee, Zihua Wang, Esra Dikoglu, Vaidehi Jobanputra, Gholson J. Lyon, Michael Wigler, Michael C. Schatz, and Giuseppe Narzisi. 2016. 'Indel variant analysis of short-read sequencing data with Scalpel', *Nature Protocols*, 11: 2529-48.

Li, H., B. Handsaker, A. Wysoker, T. Fennell, J. Ruan, N. Homer, G. Marth, G. Abecasis, and R. Durbin. 2009. 'The Sequence Alignment/Map format and SAMtools', *Bioinformatics*, 25: 2078-9.

Li, Heng. 2013. 'Aligning sequence reads, clone sequences and assembly contigs with BWA-MEM', *ArXiv*, 1303.

McKenna, A., M. Hanna, E. Banks, A. Sivachenko, K. Cibulskis, A. Kernytsky, K. Garimella, D. Altshuler, S. Gabriel, M. Daly, and M. A. DePristo. 2010. 'The Genome Analysis Toolkit: a MapReduce framework for analyzing next-generation DNA sequencing data', *Genome Res*, 20: 1297-303.

Quinlan, A. R., and I. M. Hall. 2010. 'BEDTools: a flexible suite of utilities for comparing genomic features', *Bioinformatics*, 26: 841-2.

Saunders, C. T., W. S. Wong, S. Swamy, J. Becq, L. J. Murray, and R. K. Cheetham. 2012. 'Strelka: accurate somatic small-variant calling from sequenced tumor-normal sample pairs', *Bioinformatics*, 28: 1811-7.

Wilm, A., P. P. Aw, D. Bertrand, G. H. Yeo, S. H. Ong, C. H. Wong, C. C. Khor, R. Petric, M. L. Hibberd, and N. Nagarajan. 2012. 'LoFreq: a sequence-quality aware, ultra-sensitive variant caller for uncovering cell-population heterogeneity from high-throughput sequencing datasets', *Nucleic Acids Res*, 40: 11189-201.
